# Microvascular changes in obese adults detected by Optical Coherence Tomography Angiography

**DOI:** 10.22336/rjo.2023.25

**Published:** 2023

**Authors:** Goksu Alacamli, Sema Tamer Kaderli, Sultan Edebali, Oznur Guven Alacamli, Sabahattin Sul, Tugba Dubektaş Canbek, Aylin Karalezli

**Affiliations:** * Ophthalmology Department, Trakya University Faculty of Medicine, Education and Research Hospital, Edirne, Turkey; ** Ophthalmology Department, Mugla University Faculty of Medicine, Education and Research Hospital, Mugla, Turkey; *** Internal Medicine Department, Mentese State Hospital, Mugla, Turkey; **** Diet Department, Edirne State Hospital, Edirne, Turkey; ***** Internal Medicine Department, Mugla University Education and Research Hospital, Mugla, Turkey

**Keywords:** obesity, Optical Coherence Tomography Angiography, subfoveal choroidal thickness, vessel density

## Abstract

**Aim:** The aim of this prospective, controlled, non-randomized study was the comparison of the retinal microvascular parameters of obese and nonobese adults.

**Methods:** 184 eyes of 92 subjects were separated to 3 groups. 68 eyes were in the normal weight group, with a body mass index between 18.5 and 24.5 kg/ m2, 60 eyes were in the overweight group, with a body mass index between 25-29.9 kg/ m2, and 56 eyes were in the obese group, with a body mass index ≥ 30 kg/ m2. All the volunteers were applied visual acuity, ocular motility testing, and slit lamp and mydriatic fundus examination. Optical Coherence Tomography Angiography (OCT-A) scanning was practiced with Optovue (Optovue, Inc; Fremont, CA) on a 6.00 x 6.00 mm macular region, in the central fovea.

**Results:** 184 eyes of ninety-two patients were involved in this prospective study. The vessels’ density (VD) in the optic nerve head (ONH) were significantly lower in the overweight and obese adult volunteers compared to the normal weight control group. However, other OCTA parameters (including macular VDs, Foveal avascular zone (FAZ), choriocapillaris plexus (CCP) area) did not demonstrate any significant difference between groups. Subfoveal choroidal thickness (SCT) was higher in the overweight and obese patients when compared to the normal weight control group. Central macular thickness (CMT) did not reveal any significant difference between groups.

**Conclusion:** Even though clinicians are limited in pointing out any differential findings in obese patients only by fundus examination, OCT-A provides a predictable view of the microvascular changes in the retina and choroid in obese patients.

**Abbreviations: **BMI = Body mass index, WHO = World Health Organization, AMD = Age-related macular degeneration, CT = Choroidal thickness, OCTA = Optical Coherence Tomography Angiography, (W/H) ratio = Waist-hip ratio, ETDRS = Early Treatment Diabetic Retinopathy Study, VD = Vessel density, SCP = Superficial capillary plexus, DCP = Deep capillary plexus, CCP = Flow area of the choriocapillaris, FAZ = Avascular zone, CMT = Central macular thickness, ONH = Optic nerve head

## Introduction

Obesity is a worldwide health problem in all the countries regardless of development, age, gender, and ethnicity. Approximately 10% of the 22 million children worldwide, aged < 5 years, are estimated to be overweight and obese. Body mass index (BMI) is generally used to classify obesity degree. BMI calculation is done by division of weight by the square of the height (kg/ m2). According to the World Health Organization (WHO) BMI classification system, a BMI of 18.5-24.9 kg/ m2 defines a normal body weight, a BMI of 25-29.9 kg/ m2 defines overweight, and a BMI of 30 kg/ m2 or greater defines obesity. Obesity increases the risk of cardiometabolic disorders such as atherosclerosis, nonalcoholic fatty liver disease, steatohepatitis, hypertension, and diabetes mellitus. Obesity also increases the risk of ocular pathologies associated with visual loss. These pathologies can be cataract, senile macular degeneration, glaucoma and diabetic retinopathy [**1**,**2**]. 

In several studies, high-fat intakes were found to lead to the tendency of having a high incidence of age-related macular degeneration (AMD) [**3**]. A fundus camera was used to capture the images of the retinal vessels to determine the relations between the diameter of retinal vessels and cardiometabolic risk factors in the study involving the overweight and obese children. This relation can be predictable for early microvascular impairments in these patients [**4**]. Retinal vessels have hard intrinsic autoregulatory mechanisms, whereas choroidal vessels do not. Also, in obese women, the BMI and choroidal thickness (CT) changes showed a positive correlation [**5**]. 

The success of non-invasive Optical Coherence Tomography Angiography (OCTA) in detecting disorders in microvascular circulation is undeniable. Studies in literature showed that retina-choroidal circulation is subclinically affected in many systemic diseases. An increased number of inflammatory cytokines and oxidative stress biomarkers in obese individuals has been shown in many studies, however, the correlation between obesity and retinal inflammation has yet to be studied. We aimed to compare and evaluate the retinal microvascular parameters in overweighted, obese and non-obese adults.

## Methods

This prospective study was undergone in accordance with the principles of the Helsinki Declaration and with the approval of the Ethics Committee of Mugla University Medical School of Medicine (Registration Number: E-72855364-050.01.04-238071). Patients diagnosed with obesity and healthy controls were included in the study, which lasted from October 2020 to March 2021. All the participants signed an informed consent for the study.

184 eyes of 92 adult volunteer participants were separated into 3 groups. 68 eyes were in the group of normal weighted, with a body mass index between 18.5 and 24.5 kg/ m2, 60 eyes were in the group of overweighted, with a body mass index between 25 and 29.9 kg/ m2, 56 eyes were in the obese group, with a body mass index ≥ 30 kg/ m2. Healthy non-obese adult participants, who applied to the ophthalmology clinic for a routine examination, were added to the control group.

Patients diagnosed with hypertension, diabetes mellitus, cataract, glaucoma or any retinal disease, with a history of ocular surgery or low cooperation for OCTA measurement, were not included in the study, and afterwards were excluded from this study. All the subjects underwent a detailed physical examination and medical history of all the participants was established. Also, to calculate the BMI, arterial blood pressure, weight, and height measurements were taken. Moreover, to calculate the BMI, the weight in kilograms was divided by the height in squared meters (kg/ m2). 

All the participants were applied visual acuity and ocular motility testing. They underwent slit lamp examination for anterior segment details. Also, mydriatic fundus examination was executed. Optovue (Optovue, Inc; Fremont, CA) device was used for OCT-A. 6.00 x 6.00 mm macular region centered on the fovea was the target for measurements. The parafoveal regions of the *Early Treatment Diabetic Retinopathy Study* (ETDRS) grid were used for the local examination of the vessel density (VD) for both the superficial capillary plexus (SCP) and the deep capillary plexus (DCP). Flow area of the choriocapillaris (CCP) layer was obtained at 1 mm radius areas, which was centered on the foveal avascular zone (FAZ). Fovea centered scan area of 6 mm × 6 mm was performed in this study. OCTA images with quality below 8/ 10 were excluded. Regarding the inability of automatic layer splitting, the adjustment was applied manually.

Data collected form the participants were analyzed by SPSS version 21.0.0.0 software (IBM Corp Armonk, NY, 2012). Quantitative components were tested by the Kolmogorov-Smirnov test for the normality hypothesis. Comparisons between groups were done with independent-samples t test, Kruskall Wallis test. Pearson’s correlation analyses were performed to detect the relationships between the nonperfusion area and the OCTA parameters. It was agreed that a p value lower than 0.05 was statistically significant.

## Results

184 eyes of ninety-two participants were involved in our prospective study. The demographic and clinical features are shown in **Table 1**. Groups have shown no statistically significant difference in any demographic parameter (**Table 1**). Spherical equivalent and axial length showed no statistically significant difference between groups (**Table 1**).

**Table 1:** Demographic and clinical features

	Mean ± SD			
Groups Associated with BMI, n	Group 1 25-29.9 (n=30)	Group 2 ≥ 30 (n=28)	Control < 25 (n=34)	p
Age, years	23.5 ± 3.4	24.5 ± 5.9	23.5 ± 4.4	0.388
Sex, F/ M	16/ 14	18/ 10	22/ 12	0.572
Weight, kg	74.6 ± 6.3	95.4 ± 13.8	58.1 ± 7	< 0.001
Height, cm	165.3 ± 8.0	167.1 ± 7.0	163.5 ± 7.4	0.030
BMI, kg/ m2	27.3 ± 1.2	33.9 ± 2.6	21.6 ± 1.7	< 0.001
Eyes, n	(n=60)	(n=56)	(n=68)	
Spherical equivalent, Diopters	0.35 ± 0.8	0.33 ± 0.43	0.36 ± 0.74	0.311
Axial length, mm	23.06 ± 0.49	22.84 ± 0.52	22.79 ± 0.42	0.612
SD = Standard deviation, BMI = Body mass index				

The comparison of OCT-A features between the groups was summarized (**Table 2**). The VDs in the optic nerve head (ONH) were significantly lower in both overweighted and obese patients than in the control group (**Table 2**). Whereas, there was no significant difference between groups in terms of other OCTA parameters (including macular VDs, FAZ, CCP area). SCT was higher in both overweighted and obese patients than in the control group. Central macular thickness (CMT) of the participants did not indicate any significant difference (**Table 2**).

**Table 2:** Comparison of OCT and OCTA features between obese patients and control group

		Mean ± SD					
		Group 1 25-29.9 (n=60)	Group 2 ˃ 30 (n=56)	Control < 24.9 (n=68)	pA	pB	pC
FAZ, mm2		0.224 ± 0.08	0.219 ± 0.10	0.217 ± 0.11	0.245	0.412	0.056
VDs in SCP, %	Parafovea	55.16 ± 2.1	55.46 ± 2.6	55.96 ± 2.5	0.152	0.487	0.784
	Perifovea	55.36 ± 2.3	55.67 ± 1.6	55.30 ± 2.3	0.538	0.893	0.807
	Whole	54.69 ± 1.9	53.65 ± 1.8	54.14 ± 1.8	0.621	0.428	0.550
VDs in DCP, %	Parafovea	61.52 ± 2.9	61.85 ± 2.3	61.63 ± 3.6	0.974	0.973	0.832
	Perifovea	61.63 ± 5.2	61.37 ± 4.7	62.43 ± 4.8	0.631	0.464	0.959
	Whole	60.82 ± 4.2	60.02 ± 4.8	60.91 ± 4.2	0.999	0.499	0.539
VDs in ONH, %	Peripapillary	51.25 ± 3.2	50.65 ± 2.8	53.29 ± 4.3	< 0.001	< 0.001	0.046
	Inside disc	48.20 ± 2.7	45.75 ± 3.1	51.80 ± 1.5	< 0.001	< 0.001	< 0.001
	Total disc	49.85 ± 2.2	49.3 ± 2.8	50.85 ± 1.7	0.001	0.001	0.043
CCP flow area, mm2		2.221 ± 0.10	2.199 ± 0.12	2.151 ± 0.22	0.04	0.254	0.706
CMT, µm		311.4 ± 41.3	309.8 ± 51.1	310.4 ± 63.5	0.875	0.795	0.702
SCT, µm		304.1 ± 54	315.5 ± 45.8	241.8 ± 38.6	0.04	0.019	0.153
RNFL, µm		115 ± 8.9	112.35 ± 7.7	117.3 ± 7.1	0.652	0.115	0.254
SD = Standard deviation, VD = Vessel density, SCP = Superficial capillary plexus, DCP = Deep capillary plexus, CCP = Choriocapillaris plexus, SCT = Subfoveal choroidal thickness, RNFL = Retinal nerve fiber layer							
pA, group 1 vs. control; pB, group 2 vs. control; pC, group 1 vs. group 2.							

The correlation between BMI and OCTA parameters are shown in **Table 3**. There was a mild positive correlation between SCT and BMI and no statistically significant difference was detected (**Table 3**). A positive correlation was observed between CCP flow area and BMI, but it was not statistically significant (**Table 3**). We evaluated a negative correlation between the VDs in ONH and it was statistically significant (**Table 3**). We also evaluated a negative correlation between the VDs in SCP (parafovea, perifovea and whole) and BMI, but it was not statistically significant (**Table 3**). The results showed a negative correlation between the VDs in DCP (perifovea and whole) and BMI, but it was also not statistically significant (**Table 3**). Finally, we showed a negative correlation between the RNLF and BMI, but it was not statistically significant (**Table 3**).

**Table 3:** Correlation between BMI and OCTA parameters

		r	P value
FAZ, mm2		-0.218	0.03
VDs in SCP, %	Parafovea	-0.95	0.200
	Perifovea	-0.266	0.101
	Whole	-0.160	0.130
VDs in DCP, %	Parafovea	0.37	0.617
	Perifovea	-0.53	0.475
	Whole	-0.26	0.722
VDs in ONH, %	Peripapillary	-0.628	0.001
	Inside disc	-0.574	0.001
	Total disc	-0.445	0.001
CCP flow area, mm2		0.19	0.794
SCT, µm		0.537	0.03
RNFL, µm		-0.204	0.071
VD = Vessel density, SCP = Superficial capillary plexus, DCP = Deep capillary plexus, CCP = Choriocapillaris plexus, CMT = Central macular thickness, SCT = Subfoveal choroidal thickness			

## Discussion

The aim of the study was to demonstrate that the OCTA parameters generate the overall potency for the evaluation of the microvessels in the overweighted and obese patients. The microvascular structure of ONH (prepapillary, inside disc, total disc) was influenced in overweight and obese patients. FAZ was higher in overweight and obese adult participants. However, no statistically significant difference was observed between the groups. SCT was higher in both overweighted and obese patients when compared to the control normal weight group. We obtained a mild positive correlation between SCT and BMI and a negative correlation between the VDs in ONH.

Obesity is a very common pathology, with changes in inflammatory, hormonal and metabolic factors in its pathogenesis. One of the mechanisms that may be the basis of ocular diseases common with obesity, can be disruptions in the retinochoroidal microvascular system [**1**]. Obesity causes microvascular changes in many organ systems. In obesity, it has been shown that the venules are enlarged because of the increased fat tissue, and microvascular dysfunction may be present in its etiology. In the study undergone, we compared the retinochoroidal vascular parameters in obesity patients with normal healthy individuals. 

It may cause impairment of the metabolic profile in obese patients due to systemic oxidative stress secondary to hyperleptinemia. Some studies showed that nitric oxide (NO) levels decreased and vessels widened in obese individuals. There is a marked association between obesity and metabolic syndrome, which is related to a proinﬂammatory and proatherogenic circumstance that causes a tendency to cardiovascular disease. Although the initial mechanism of atherosclerosis is still not known, inﬂammation and endothelial function deterioration seem to be key events. These vascular changes can also occur in the eye, which is an end organ, and may result in decreased vascular flow to the retina. Reduced oxygenation and flow may be the reason for the development of vascular diseases of the retina. Thus, quantitative parameters related to OCTA can provide important information regarding subclinical changes occurring in obese participants. In literature, it has been demonstrated that this relationship may be due to systemic oxidative stress secondary to hyperleptinemia, which is caused by obesity. In previous studies, obesity has been indicated as a risk factor for retinal vascular diseases [**6**,**7**]. 

Other studies about obese patients have shown that choroidal thickness increases in obese subjects when compared to the healthy control population [**8**,**9**]. In our study, similar to their results, we observed higher choroidal thickness values compared to the healthy controls. Choroidal thickness values and pathological changes in obese participants were shown in another study with OCTA [**9**]. Also, the authors of the same study indicated that arteriovenous ratio values were lower than normal in control participants. All these data showed that both retina and choroidal microvasculature can be negatively affected in obesity.

Zarei et al. found that the RNFL thickness of the optic disc is lower in adult individuals with metabolic syndrome, compared to the controls [**10**]. Another study mentioned that both retinal blood flow and retinal thickness were lower in diabetic obese mice, compared to non-obese mice [**11**]. In the same study, although there was no significant decrease in retinal VD, a significant retinal blood flow was detected. Icel et al. reported that the RNFL thickness was lower in the obese adult patients than in the non-obese group, but they did not find it statistically significant. In another study involving children, a reduction in RNFL due to obesity was determined [**12**]. They found that the RNFL was negatively correlated with the BMI. We observed a negative correlation between the RNFL and BMI, but no statistical significance was found, similar to the studies mentioned above. 

OCT-A provides an understanding of both the retinal and the choroidal microvascular structure. By OCT-A, worthwhile data with structural features of the retina can be obtained. It is an effective and useful imaging method that can detect the changes in initial phases in which the retina is affected.

The VDs in ONH were significantly lower in the overweighted and obese patients than in the control group (**Table 2**). This difference can be a predictor of pre-clinic pseudotumor cerebri. We could not find any article or study showing ONH vessel density in obese patients. Thus, we could not compare our ONH VD results to any other results.

According to Zhi Z et al. [**11**], Agarwall A et al. [**9**], Icel E et al. [**13**], no significant difference regarding the parafoveal and perifoveal vessel density was observed in any of the groups. The study of Icel E et al. was performed only in women, the one of Zhi Z et al. only in mice, and the one of Agarwall A et al. only in men. But, in our study, patients of both sexes were involved in the study, homogeneously. According to Zhi Z et al. [**11**], Agarwall A et al. [**9**] and Icel E et al. [**13**], even though there was no statistical significance, a negative correlation was obtained between the VDs in SCP (parafovea, perifovea and whole) and BMI and the VDs in DCP (perifovea and whole) and BMI.

Although there was no statistical significance, Icel E et al. [**13**] also found a positive correlation between the FAZ and BMI, in contrast to our study. Icel E et al. [**13**] performed their study only in women, but in our study, the sex of patients was homogeneous. Icel E et al. [**13**] observed a negative correlation between the RNLF and BMI, but they did not detect a statistical significance, which was similar to our study. 

## Conclusion

OCT-A is a new method for imaging the retinal microvasculature. Our study is the first one in literature to have included homogeneous sex and BMI groups and to have indicated the microvascular changes in obese patients. Despite that, we did not detect any significant difference in all the groups matched with their demographic parameters in our study. Moreover, more studies are required on this subject with a higher number of cases.

Even though clinicians are limited in pointing out any differential findings in the obese patients only by fundus examination, OCT-A provides an understanding of the retinal and choroidal microvascular changes in these patients.

**Conflict of Interest statement**

The authors state no conflict of interest.

**Informed Consent and Human and Animal Rights statement**

Informed consent has been obtained from all individuals included in this study.

**Authorization for the use of human subjects**

Ethical approval: The research related to human use complies with all the relevant national regulations, institutional policies, is in accordance with the tenets of the Helsinki Declaration, and has been approved by the Ethics Committee of Mugla University Medical School of Medicine, Mugla, Turkey (Registration Number: E-72855364-050.01.04-238071).

**Acknowledgements**

None.

**Sources of Funding**

None.

**Disclosures**

None.
